# Deterioration in cognitive control related mPFC function underlying development of treatment resistance in early psychosis

**DOI:** 10.1038/s41598-024-63474-1

**Published:** 2024-06-06

**Authors:** Charlotte M. Crisp, Angad Sahni, Sze W. Pang, Lucy D. Vanes, Timea Szentgyorgyi, Bruno Averbeck, Rosalyn J. Moran, Sukhwinder S. Shergill

**Affiliations:** 1 School of Psychological Sciences, University of Bristol, 12a Priory Road, Bristol, BS8 1TU UK; 2 Institute of Psychiatry, Psychology and Neuroscience, King’s College London, De Crespigny Park, London, SE5 8AF UK; 3 Laboratory of Neuropsychology, National Institute for Mental Health, Bethesda, Bethesda, MD 20814 USA; 4 Kent and Medway Medical School, University of Kent, Parkwood Road, Kent, CT2 7FS UK

**Keywords:** Cognitive control, Treatment resistance, Schizophrenia, fMRI, Glutamate, Spectroscopy, Predictive markers, Brain imaging, Reward, Cognitive control, Psychosis

## Abstract

One third of people with psychosis become antipsychotic treatment-resistant and the underlying mechanisms remain unclear. We investigated whether altered cognitive control function is a factor underlying development of treatment resistance. We studied 50 people with early psychosis at a baseline visit (mean < 2 years illness duration) and follow-up visit (1 year later), when 35 were categorized at treatment-responsive and 15 as treatment-resistant. Participants completed an emotion-yoked reward learning task that requires cognitive control whilst undergoing fMRI and MR spectroscopy to measure glutamate levels from Anterior Cingulate Cortex (ACC). Changes in cognitive control related activity (in prefrontal cortex and ACC) over time were compared between treatment-resistant and treatment-responsive groups and related to glutamate. Compared to treatment-responsive, treatment-resistant participants showed blunted activity in right amygdala (decision phase) and left pallidum (feedback phase) at baseline which increased over time and was accompanied by a decrease in medial Prefrontal Cortex (mPFC) activity (feedback phase) over time. Treatment-responsive participants showed a negative relationship between mPFC activity and glutamate levels at follow-up, no such relationship existed in treatment-resistant participants. Reduced activity in right amygdala and left pallidum at baseline was predictive of treatment resistance at follow-up (67% sensitivity, 94% specificity). The findings suggest that deterioration in mPFC function over time, a key cognitive control region needed to compensate for an initial dysfunction within a social-emotional network, is a factor underlying development of treatment resistance in early psychosis. An uncoupling between glutamate and cognitive control related mPFC function requires further investigation that may present a future target for interventions.

## Introduction

Antipsychotic treatment resistance affects approximately one third of people with schizophrenia^1^. There is an urgent need to understand the underlying neurobiological mechanisms in order to identify people at risk of treatment resistance and to develop targeted treatment strategies and new medications to improve prognosis^2^. One mechanism of interest that could help us to understand treatment-resistant schizophrenia is cognitive control. Cognitive control (or ‘executive control’) refers to processes that support flexible responses and goal-directed behaviour^3^. Alterations in cognitive control and prefrontal cortex function (supporting cognitive control) or ‘hypofrontality’ have been emphasized in the schizophrenia literature, however it is unknown whether these deficits relate to treatment resistance. In chronic schizophrenia, previous studies have shown that treatment-resistant participants may have intact dopaminergic function^4^, including reward prediction error (RPE) signaling^5^, but higher levels of glutamate in anterior cingulate cortex (ACC)^4,6^ compared to treatment-responsive participants. In our previous fMRI connectivity study using an emotion-yoked reward learning task that required cognitive control, we suggested that the persistence of positive symptoms occurs when a cognitive control mechanism that was needed to contextualize aberrant salience during reward learning was impaired^7^. In treatment-responsive participants with chronic schizophrenia, there was elevated top-down connectivity from ACC to sensory regions (fusiform gyrus and amygdala) and lower connectivity from all three regions into striatum. This was interpreted as a compensatory mechanism allowing abnormal experiences, and positive symptoms, to be controlled. In contrast, treatment-resistant participants with chronic schizophrenia lacked this compensatory cognitive control mechanism and top-down connectivity was also uncoupled to ACC glutamate levels^7^. This suggests a subtype of schizophrenia with a potential primary non-dopaminergic mechanism that is not targeted by currently available medication.

In early psychosis, it is unclear whether a cognitive control dysfunction exists from the first episode and predicts future treatment resistance or if cognitive control deteriorates over time leading to treatment resistance. Two studies in unmedicated people with schizophrenia used a typical cognitive control task (Stroop colour task) to investigate which regions are most important for initial treatment response. The authors found that modulation of cognitive control function was important for a good initial improvement in symptoms in response to a 6-week course of medication^8,9^. However, lower activity in reward structures (striatum and midbrain) at baseline best predicted a better treatment response. Recently, we studied the same emotion-yoked reward learning task that requires cognitive control (reported above) in medicated early psychosis participants with greater severity of positive symptoms. We showed that these participants had dysfunction within a social-emotional network including amygdala, pallidum and thalamus, and a cognitive control region (lower activity in supplementary motor area (SMA)) compared to early psychosis participants with lower severity of positive symptoms^10^. Taken together, these studies suggest a primary alteration within a subcortical network is present in both medicated and unmedicated people with early psychosis and that inadequate compensatory mechanisms may exist in this early stage of illness which are related to a poorer initial response to antipsychotics and greater severity of positive symptoms.

The aim of the present study was to understand factors leading to treatment resistance in early psychosis. We present data from a longitudinal multimodal study that compared changes in brain activity relating to cognitive control function during the same emotion-yoked reward learning task (reported above) and ACC glutamate levels between 50 early psychosis participants that became treatment-resistant or treatment-responsive from baseline to one-year follow-up. We chose the emotion-yoked reward learning task because we wanted to study reward processing (shown to be abnormal in schizophrenia^11^ but intact in treatment-resistant schizophrenia^5^) alongside cognitive control (shown to be impaired in treatment-resistant schizophrenia^12^) as a way to study these functions concurrently. The task requires cognitive control because it elicits an emotional bias that requires participants to flexibly overcome their prepotent response towards positive emotions and direct their choices towards the objective feedback they receive instead. Moreover, we chose to study glutamate levels in the ACC using MRS because some evidence suggests that treatment-resistant people have elevated glutamate in this region which could be a potential non-dopaminergic treatment target^4^. However, very few studies have investigated glutamate longitudinally or in relation to cognitive control related activity and so we sought to compare how ACC glutamate levels change over time in relation to cognitive control related function between people that became treatment-resistant and treatment-responsive. Three hypotheses were evaluated: (1) treatment-resistant participants would show decreased cognitive control related activation in ACC/medial prefrontal cortex (mPFC) from baseline to 1-year follow-up accompanied by increased activation in social-emotional regions (amygdala, pallidum, thalamus) compared to treatment-responsive participants, (2) ACC/mPFC activity would be uncoupled to ACC glutamate levels in treatment-resistant participants^7^, and (3) differences in social-emotional region activity at baseline would be predictive of treatment resistance status at follow-up^8^.

## Methods and materials

### Participants

**Figure 1:**
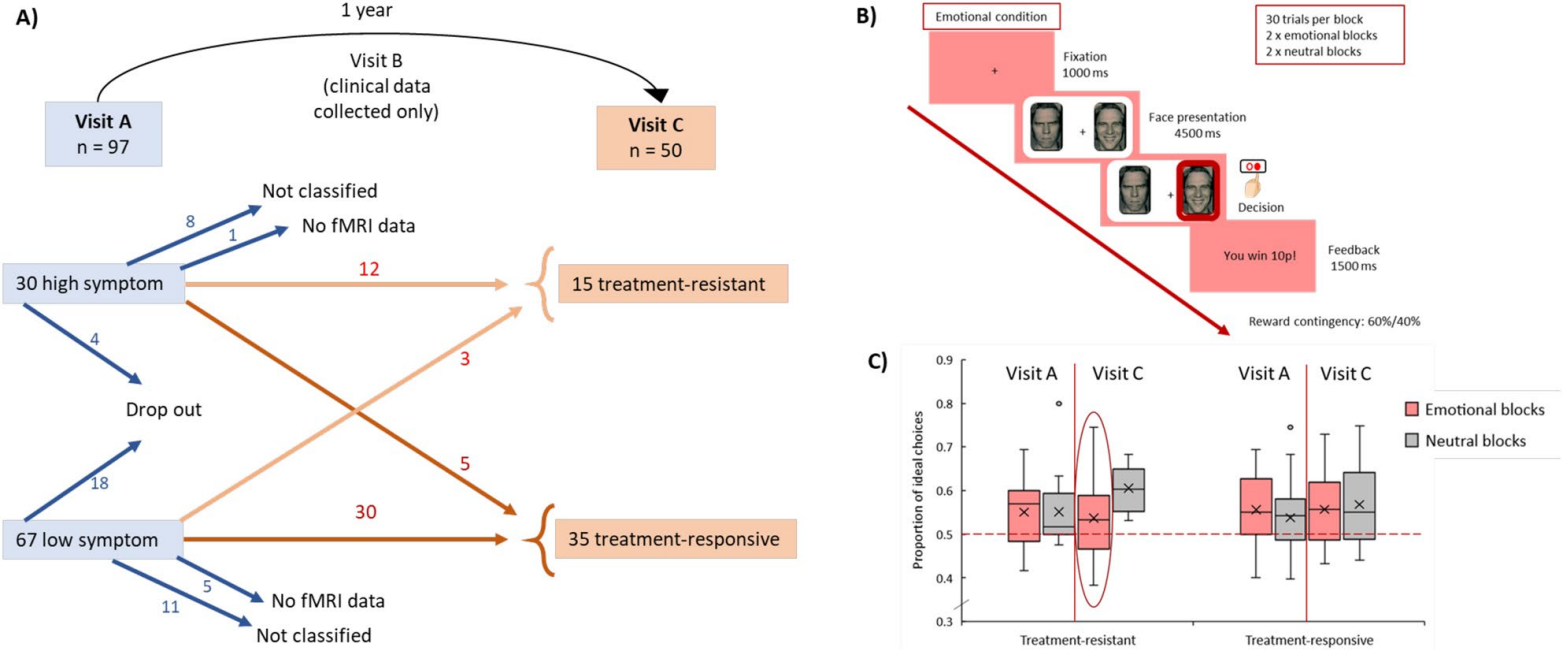
Participant trajectories and reward learning behaviour. (**A**) Number of participants that were classified as having high and low positive symptoms (reported in Horne et al.^10^) at visit A (baseline) and the trajectories of those participants one year later at visit C (final follow up). The final sample included here consists of 15 treatment-resistant and 35 treatment responsive participants with fMRI data from both visits. (**B**) A diagram of the fMRI task where participants learnt which facial expression was associated with a higher probability of reward (60/40% contingencies). (**C**) Box plot showing the mean proportion of ideal choices made in each condition (emotional/neutral) and at each visit (A/C) by treatment-resistant and treatment-responsive participants. All participants perform significantly above chance level (red dashed line at 0.5) for each condition (one-sample t-tests) except for emotional blocks at visit C where treatment-resistant participants do not perform significantly above chance (circled in red). There is a significant visit x condition interaction (more ideal choices made in neutral vs. emotional conditions at visit C).

The study included 50 people with early psychosis recruited from the South London and Maudsley (SLaM) NHS foundation trust, and from the Oxleas NHS Foundation Trust and North East London NHS Foundation Trust (NELFT). Participants attended three study visits: visit A (baseline), visit B (six month follow-up) and visit C (one year follow-up) where fMRI data was collected at visits A and C, and clinical assessments were done at all three visits. Only participants that had fMRI data from both visits (A and C) were included (see Fig. 1A for information about patient trajectories). We included participants that experienced a first episode of psychosis at visit A within the last 5 years (mean illness duration = 1.6 years); and categorised 15 as ‘treatment-resistant’ and 35 as ‘treatment-responsive’ at visit C. Categorisation was determined based on strict criteria for defining treatment-resistant schizophrenia and according to TRRIP recommendations^13^. Participants were categorised as treatment-resistant if they met the symptom threshold at visit C (at least one positive symptom item of five (moderate severe) or higher, or at least two positive symptom items of four (moderate) or higher measured using the Positive and Negative Syndrome Scale (PANSS)^13^, had undergone at least two adequate medication trials (4–6 week duration at 400–600 mg of chlorpromazine or equivalent)^14^, had overall adequate medication adherence and had low social-occupational functioning (score below 60) as defined using the Social and Occupational Functioning Assessment scale (SOFA)^15^. Participants were categorised as treatment-responsive if they had an overall adequate medication adherence and did not meet the symptom threshold at visit B or C—ensuring 6-month symptom stability. Participants that did not meet the criteria for either treatment-resistance or treatment-responsive (e.g., were not medication adherent, had not completed adequate medication trials, had treatment-resistant symptoms but a high SOFA score) were not classified and were excluded from analyses. Additional assessment and medication details for categorisation can be found in the “supplementary materials”.

The Camberwell and St. Giles NHS National Research Ethics Committee granted ethical approval and the study complied with the Declaration of Helsinki. All participants provided written informed consent prior to participating and were compensated for their time and travel.

### Reward learning task

All participants underwent an emotion-yoked reward learning task that requires cognitive control whilst undergoing fMRI scanning at visits A and C. An example trial sequence is shown in Fig. 1B and has been reported previously^5,7^. Participants were asked to choose between two faces (side-by-side) on each trial and were required to learn—over a series of 30 iterative trials (per block)—which of the two faces was associated with a higher probability of reward (reward contingencies were 60/40). There were two ‘emotional’ blocks, where participants chose between happy and angry facial expressions (with the same identity), and two ‘neutral’ blocks where participants chose between two neutral faces of different identities (total of four blocks). Cognitive control is required in this task because the two ‘emotional’ blocks create an emotional bias (described below) which mean participants must flexibly overcome their prepotent response towards positive emotions and direct their choices towards the objective feedback they receive instead. Structural and functional MRI data were acquired from a 3 T GE Excite 11 MR scanner (GE Healthcare, Chicago, IL). Task details are available in “supplementary materials”.

### Behavioural analysis

#### Ideal choices

Learning behaviour was assessed using a ‘double update’ reinforcement learning model (as reported previously^5,16^). The participant’s choice on each trial was classified as ‘ideal’ when the expected reward for the chosen face (Q1(t), estimated using the model) was greater than the expected reward for the unchosen face (Q2(t)). The first trial was always considered ideal. Ideal choices therefore represent how well the participant estimates the value representation of each face and translates it into their choice action. The proportion of ideal choices was computed for each condition (emotional, neutral) and each visit (A and C), excluding missing trials.

Behavioural analyses were conducted in SPSS version 25. First, eight one-sample t-tests (two conditions, two visits, two groups) were performed to test whether the proportion of ideal choices made in each group (responsive, resistant) were, on average, significantly above chance level (0.5) as an index of learning. An adjusted p-value of 0.00625 (i.e., 0.05/8) was used to correct for multiple comparisons (Bonferroni correction). Next, a 2 × 2 × 2 mixed model ANOVA was used to analyse interactions between groups and visits in the proportion of ideal choices made.

#### Emotional bias

Similar to our previous papers^5,7^, an overall ‘emotional bias’ score was calculated for each participant during the emotional condition. The score indicates the degree of bias towards choosing happy over angry faces which has shown to be different in schizophrenia^17^. It is calculated as the difference between the proportion of happy faces chosen given the angry face was determined as ideal, and the proportion of angry faces chosen given the happy face was determined ideal. A mixed-model ANOVA was used to test for a group x visit interaction on emotional bias scores.

### fMRI Imaging

#### Scanning parameters

A rapid acquisition gradient echo sequence was used to acquire T_1_-weighted images (structural images) (T_R_ = 7321 ms, T_E_ = 3 ms, T_I_ = 400 ms, field of view = 240 × 240 mm^2^, slice thickness = 1.2 mm, 196 slices). A T_2_* echo planar sequence sensitive to BOLD contrast was used to acquire functional MRI scans (T_R_ = 2 s, T_E_ = 35 ms, field of view = 24 cm, slice thickness = 3 mm, matrix = 64 × 64, flip angle = 75°, 430 volumes).

#### fMRI data analysis

The fMRI data at both visits were pre-processed and analysed in Statistical Parametric Mapping, version 12 (SPM12, available at http://www.fil.ion.ucl.ac.uk/spm/software/spm12, Wellcome Department of Cognitive Neurology, London, England) using the same procedure (see “supplementary materials”). In brief, functional images were realigned, co-registered to structural scans, normalised to Montreal Neurological Institute (MNI) space, spatially smoothed and subjected to first-level general linear model estimation where three phases of the task (face presentation, decision (button press) and feedback) were modelled for each condition (emotional, neutral) and each visit (A, C). Six contrasts of interest were constructed: (1) Emotional decisions (visit A—visit C), (2) Emotional feedback (visit A—C), (3) Neutral decisions (visit A—visit C), (4) Neutral feedback (visit A—visit C), (5) Emotional vs neutral decisions (visit A—visit C), and 6) Emotional vs. neural feedback (visit A—visit C). The six contrasts of interest were then submitted to separate group-level analyses. Two independent sample t-tests were used to compare activity between treatment-resistant and treatment-responsive participants (giving the group x visit interaction). Post-hoc tests restricted to the significant region were used to clarify the interaction effect.

#### Region of interest analyses

Analyses were conducted using three a-priori regions of interest (ROIs) based on previous literature^18–20^ and findings from the same task reported in an independent sample of people with chronic schizophrenia^7^ and from a subset of the visit A data^10^. The ROIs were (1) bilateral amygdala (decision phase), (2) bilateral subcortical structures (including striatum (caudate nucleus, putamen, nucleus accumbens) and pallidum and thalamus) (feedback phase) and (3) Anterior Cingulate Cortex (ACC) (decision and feedback phases). See “supplementary materials”. Significant effects are reported if they survived small volume correction (SVC) with a peak level threshold of *p* < 0.05 FWE-corrected. Whole-brain analyses are also reported if statistically significant (cluster-level FWE-corrected threshold of *p* < 0.05).

### Glutamate

MR spectroscopy (1H-MRS) was acquired using a standard GE PROBE (proton brain examination) sequence (Point Resolved Spectroscopy (PRESS) (T_R_ = 3000 ms, T_E_ = 30 ms, 96 averages). Before each scan, an auto-prescan was performed in order to optimise shimming and initial localiser scans and structural images were acquired in order to locate the Anterior Cingulate Cortex (ACC). Axial 2d T2-weighted fast spin echo scans and axial fast fluid-attenuated inversion recovery scans were acquired for this. A voxel measuring 20 × 20 × 20 mm was manually placed over the ACC in each participant by locating the centre of the voxel using the midline sagittal localiser slice and placing the voxel 13 mm above the genu of the corpus callosum, perpendicular to the AC-PC line. Glutamate was the primary metabolite of interest and was referenced to total Creatine (Glu/tCr). Only metabolite concentration estimates with a Cramer-Rao lower bound (CRLB) < 20%, a signal-to-noise ratio > 10 and a linewidth of FWHM < 0.1 ppm were included in analyses. Spectra were analysed using LCModel (version 6.3 http://s-provencher.com/lcmodel.shtml^21^) using a standard basis set of 16 metabolites (comprising L-alanine, aspartate, creatine, phosphocreatine, GABA, glucose, glutamine, glutamate, glycerophosphocholine, glycine, myoinositol, L-lactate, N-acetyl aspartate, N-acetylaspartylglutamate, phosphocholine, taurine), including simulated lipids and macromolecules that were acquired with the same field strength (3 T), localization sequence (PRESS) and echo time as in this study (http://www.s-provencher.com/pub/LCModel/manual/manual.pdf). Partial volume correction was not performed as concentration ratios are less sensitive to these effects. Glutamate was chosen as the primary metabolite of interest given the potential role of glutamatergic dysfunction in the development of treatment-resistant schizophrenia, although Glutamine and Glx are also reported. Glutamate levels were examined and were expressed as a ratio to total Creatine (Creatine + Phosphocreatine). First, a mixed-model ANOVA was conducted to test for a group x visit interaction on glutamate levels^4,22^. Then, we tested (1) whether glutamate (from visit A) was related to a significant change in PFC activity over time during emotional feedback, and (2) whether glutamate levels at visit C were related to PFC activity at visit C (see “supplementary materials” for details).

### Prediction

A post-hoc prediction model was constructed to test which of the regions identified in the analysis above was most predictive of treatment resistance at follow-up. Five variables of interest from visit A—right amygdala, left pallidum and mPFC activity, ACC glutamate levels and behaviour (ideal choices)—were entered into a logistic regression model to test their utility to predict treatment resistance at visit C (see “supplementary materials”).

## Results

Demographic and clinical information is reported in Table 1. Groups did not significantly differ in age, sex, age of psychosis onset, illness duration or medication dose. Treatment-resistant participants had significantly higher scores on all PANSS symptom dimensions (positive, negative, general), compared to treatment-responsive participants, and symptoms significantly increased over time.

**Table 1:** Table of demographics. Means (M) and standard deviations (SD) of key demographic and clinical information are presented for each group (treatment-resistant and treatment-responsive participants) and where appropriate, each visit.

Demographics	Treatment-resistant (n = 15)				Treatment-responsive (n = 35)						Group statistics			
	M	SD			M	SD			Stat	Sig				
									t (df)	*p*				
Age	27.2	5.8			27.3	6.8			− 0.028 (48)	0.978				
									χ^2^(df)	*p*				
Sex (number of males)	11				25				0.019 (1)	0.891				
									t (df)	*p*				
Age of onset (years)	25.3	6.1			25.8	6.8			− 0.242 (48)	0.81				
Illness duration (years)	1.9	1.1			1.4	1.2			1.521 (48)	0.135				

	Visit A		Visit C		Visit A		Visit C		Main effect of group		Main effect of visit		Group x visit interaction	
	M	SD	M	SD	M	SD	M	SD	F (df)	*p*	F (df)	*p*	**F (df)**	*p*
PANSS Positive	16.4	4.9	20.9	4.9	10.8	4.4	9.1	2.3	66.7 (1,47)	< 0.001 ***	4.3 (1,47)	0.043 *	21.5 (1,47)	< 0.001 ***
PANSS Negative	16.4	6.1	17.9	5.8	12.7	4.8	11.6	5.3	9.65 (1,47)	< 0.01 **	0.01 (1,47)	0.94	3.56 (1,47)	0.065
PANSS General	32.8	6.6	39.1	7.6	26.7	7.5	11.6	5.3	33.31 (1,47)	< 0.001 ***	0.78 (1,47)	0.38	24.81 (1,47)	< 0.001 ***
PANSS Total	65.6	14.6	77.9	13.6	50.2	15	43.4	11.3	40.04 (1,47)	< 0.001 ***	1.90 (1,47)	0.17	27.57 (1,47)	< 0.001 ***
Medication dose (CPZ equivalent)	236.4	131.9	252	86.5	229.1	119	205.9	109.3	0.55 (1,29)	0.46	0.045 (1,29)	0.83	0.88 (1,29)	0.35

Note that medication dose information was not available for 3 participants at visit A and 19 participants at visit C (14 participants were discharged and not on medication and 5 had unknown medication status). CPZ = chlorpromazine, df = degrees of freedom, PANSS = Positive and Negative Syndrome Scale. Asterisks highlight significant differences (* = *p* < 0.05, ** = *p* < 0.01, *** = *p* < 0.001).

### Behavioural results

#### Ideal choices

Treatment-responsive participants made, on average, ideal choices that were significantly above chance level for each condition and each visit (*p* < 0.001). Treatment-resistant participants showed a similar pattern of behaviour, except for the emotional condition at visit C where ideal choices were not significantly above chance (M = 0.54, S.D. = 0.094) (*p* > 0.05) (Fig. 1C).

There were no significant main effects of visit, condition, or group on the proportion of ideal choices. There was a significant visit x condition interaction (F(1,48) = 6.68, *p* = 0.013, η^2^ = 0.12), indicating that all participants performed better in the neutral compared to emotional condition at visit C (paired sample t-test t(49) = − 2.39, *p* = 0.021)) (Fig. 1C). There was no group x visit interaction, or group x condition x visit interaction.

#### Emotional bias

On average, participants showed an emotional bias towards choosing happy over angry faces (M = 0.069, S.E. = 0.014). However, scores did not significantly differ depending on group, visit or group x visit interaction (*p*’s > 0.05).

### Neuroimaging results

#### Emotional condition

**Figure 2:**
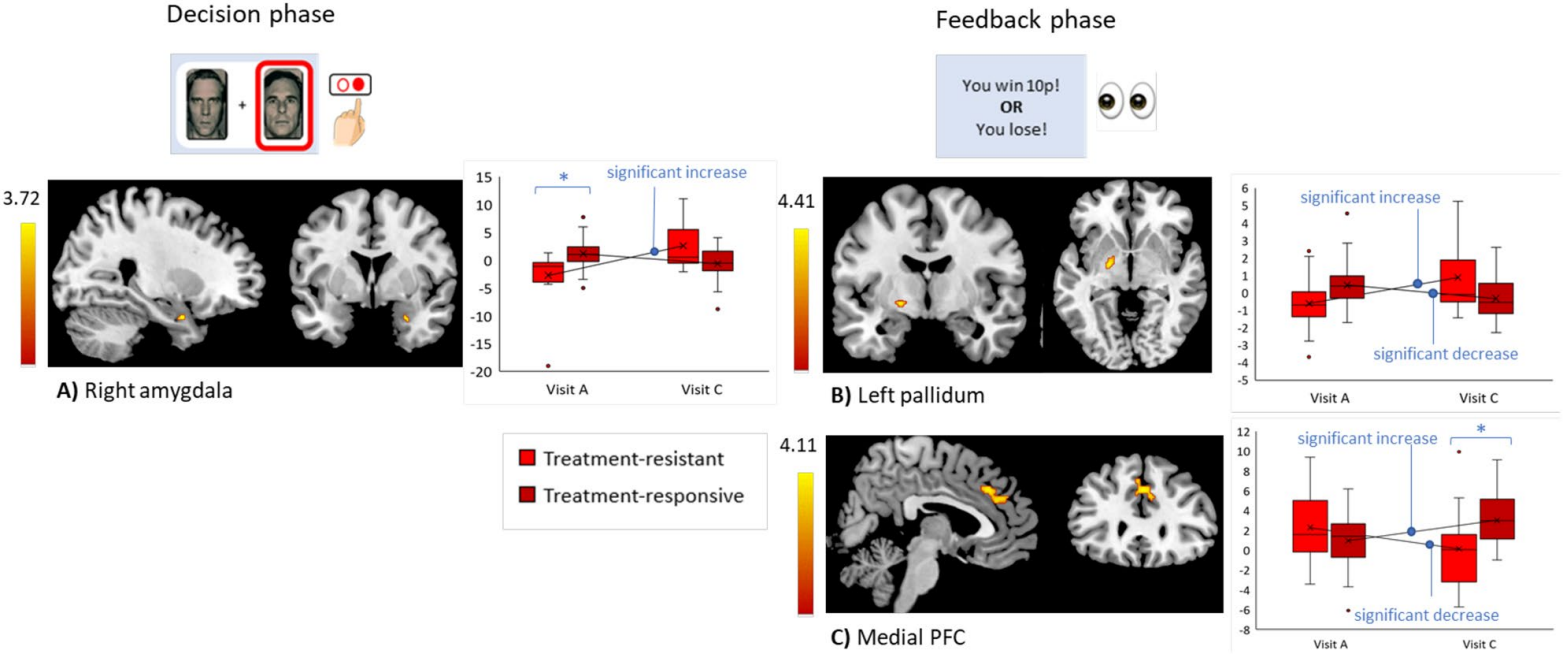
Significant group x visit interactions during the emotional condition. The figure shows three regions showing a significant group x visit interaction: (**A**) right amygdala (decision phase), (**B**) left pallidum (feedback phase), and (**C**) medial Prefrontal Cortex (mPFC) (feedback phase). Vertical colour bars indicate the associated T values. Bar charts (right) show the associated parameter estimates from the peak voxel extracted from visits A and C for each group (red = treatment-resistant, dark red = treatment-responsive) where the mean values are connected by a solid black line to show the direction of change. Post-hoc tests showing significant changes over time are indicated and the horizontal lines above boxes indicate between-group differences at each visit separately (* = *p* < 0.05 FWE-corrected).

In the decision phase, ROI analyses showed a significant group x visit interaction in the right amygdala (peak at 26, 2, − 26; T = 3.72, *p* < 0.05 small volume corrected) (Fig. 2A). Treatment-resistant participants had lower right amygdala activity at visit A compared to treatment-responsive participants (but no difference at visit C) and showed a significant increase in activity from visit A to C (whilst there was no activity change in treatment-responsive participants). There were no significant whole-brain differences.

In the feedback phase, ROI analyses showed a significant group x visit interaction in left pallidum (peak at − 20, − 6, − 4; T = 4.41, *p* < 0.05 small volume corrected) (Fig. 2B). Treatment-resistant participants had left pallidum activity that significantly increased over time whilst treatment-responsive showed a significant decrease. There were no between-group differences at visits A and C separately. Whole-brain analyses also revealed a significant group x visit interaction in medial prefrontal cortex (mPFC) (peak at 2, 30, 36; 185 voxels; T = 4.11, *p* < 0.05 cluster-level FWE-corrected) (Fig. 2C). Here, treatment-resistant participants had mPFC activity that significantly decreased over time whilst responsive participants showed a significant increase. Treatment-resistant participants also had significantly lower activity at visit C compared to treatment-responsive participants (but no differences at visit A).

#### Neutral condition

In the decision phase, ROI analyses showed a similar significant group x visit interaction in right amygdala (peak at 34, 4, − 20; T = 3.41, *p* < 0.05 small volume corrected) (Figure S1). Again, treatment-resistant participants displayed lower activity in this region at visit A compared to treatment-responsive but a significant increase in activity over time. There were no significant effects between groups using whole-brain analyses or during the feedback phase.

#### Emotional versus neutral contrast

There were no significant differences between groups for the decision phase or using ROI analyses.

For the feedback phase, whole-brain analyses showed there was a significant group x visit interaction in a similar region of the mPFC extending superiorly into supplementary motor area (SMA) (peak -4, 10, 58; 253 voxels; T = 4.49, *p* < 0.05 cluster-level FWE-corrected) (Figure S2).

### Glutamate

**Figure 3:**
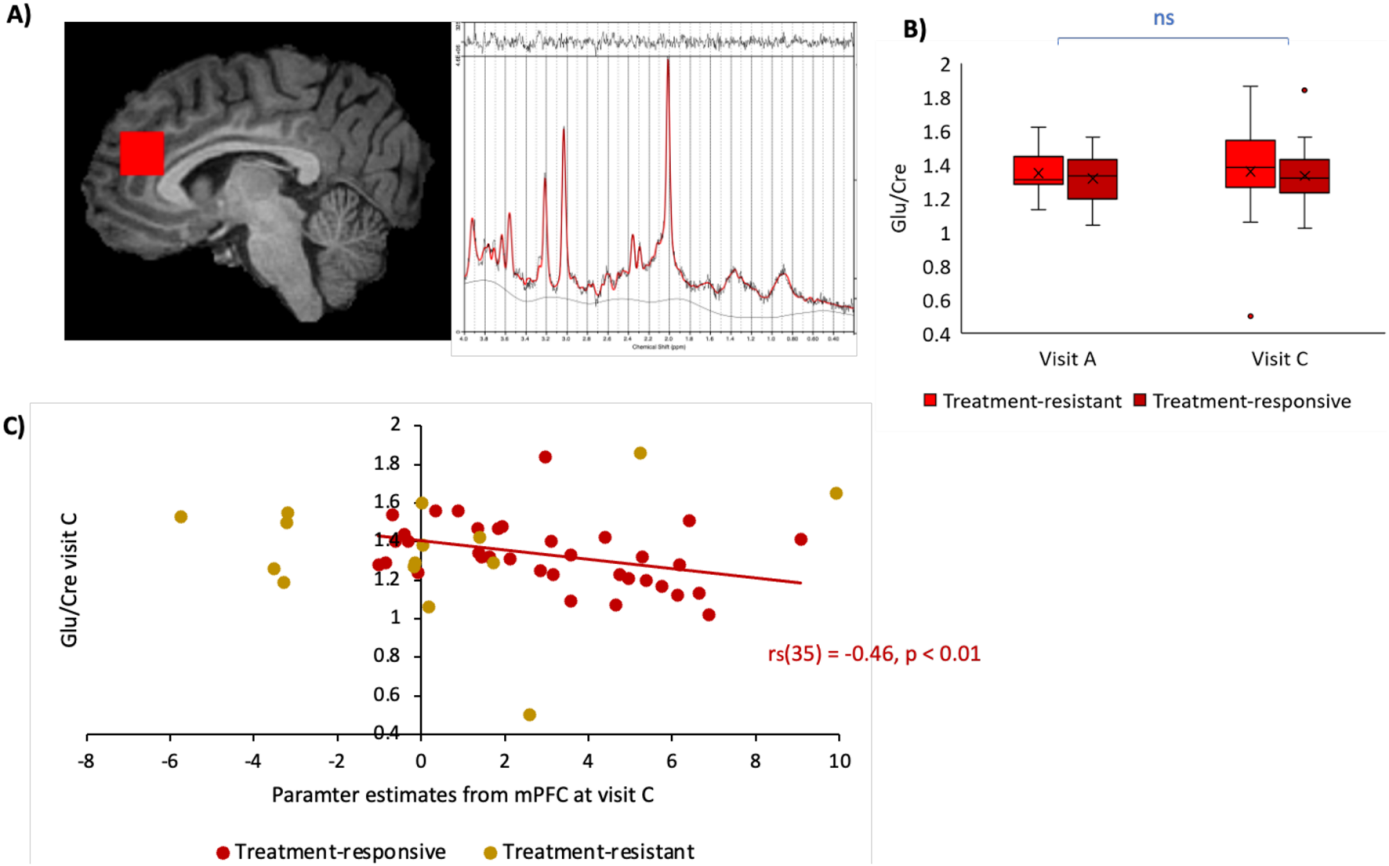
Glutamate. (**A**) An example placement of a Magnetic Resonance Spectroscopy (MRS) voxel over the Anterior Cingulate Cortex (ACC) where glutamate levels (referenced to Creatine) are measured from. (**B**) Box plot showing no significant main effect of group or visit, or group x visit interaction on glutamate levels alone. (**C**) Scatterplot showing a significant negative correlation between parameter estimates from mPFC at visit C and glutamate levels at visit C in treatment responsive participants only (*p* < 0.01). There was no significant correlation within the treatment resistant group and correlation coefficients were significantly different between groups (Fisher’s Z = − 2.006, *p* < 0.05).

Mean glutamate levels between groups and visits are presented in Table S1 along with Glx, NAA (N-acetyl aspartate + N-acetylaspartylglutamate), Myo-Inositol and Total Creatine. Spectra quality was good, especially for the primary metabolite of interest Glutamate, and there were no differences in spectra quality measures between groups or visits (Measures including CRLB%, S/N and FWHM can be found in Table S2). Glutamate levels were not significantly different between groups or visits and there was no significant group x visit interaction (Fig. 3A,B). There was also no relationship between glutamate levels at visit A and change in activity over time in mPFC (emotional feedback). A paired t-test showed that there was no statistically significant change in Total Creatine levels from visit A to C (t(49) = 1.00, *p* > 0.05) suggesting the reference metabolite was stable over time.

Across all participants, there was no significant correlation between glutamate levels and mPFC activity at visit C. Within each group separately however, there was a significant negative correlation in the treatment-responsive group (r_s_(35) = − 0.46, *p* = 0.005) (Fig. 3C) but a lack of significant correlation in the treatment-resistant group (r_s_^15^ = 0.18, *p* > 0.05). A Fisher’s Z test confirmed the correlation coefficients were significantly different between groups (Z = − 2.006, *p* < 0.05) (Fig. 3C).

### Prediction

**Figure 4:**
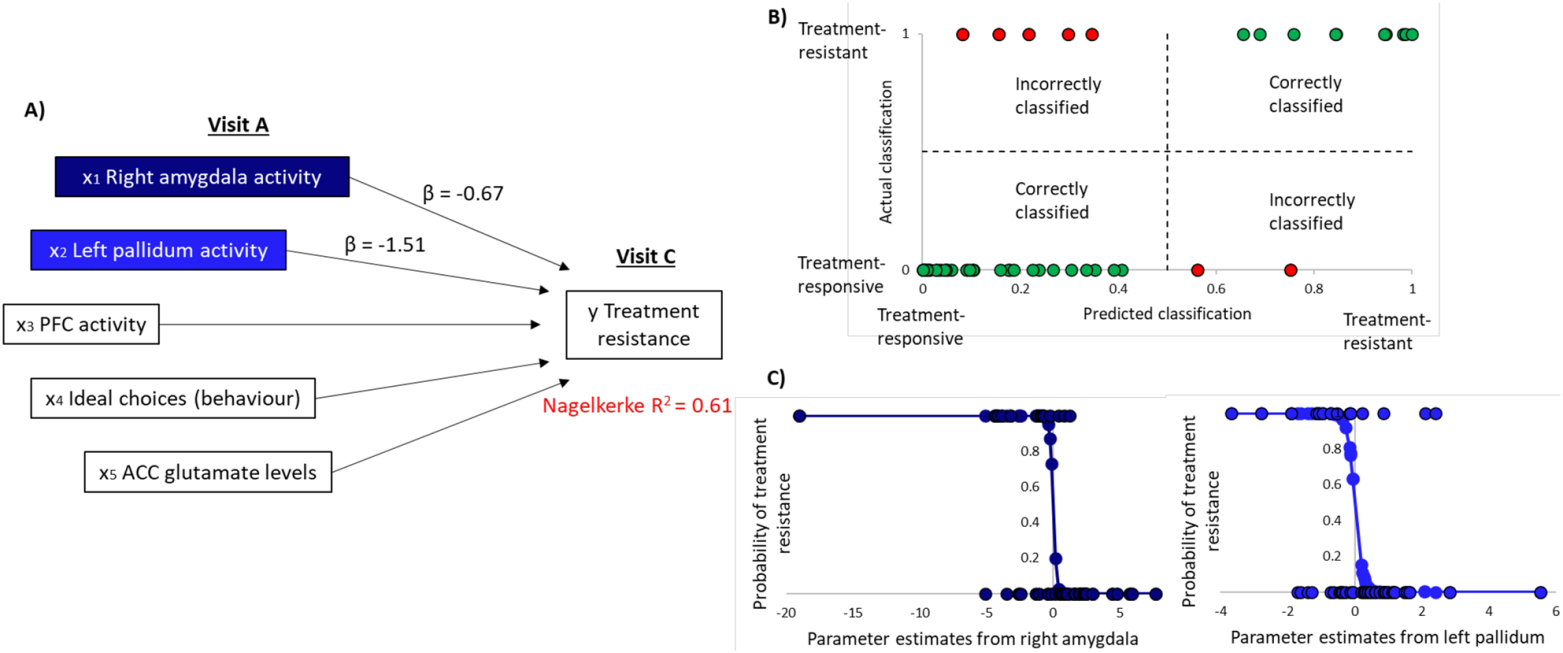
Predictors of treatment resistance. (**A**) shows the independent variables in the logistic regression model (taken from visit A) that explain 61% of the variation in treatment resistant status (at visit C). (**B**) The model correctly classified 86% of patients where 2 treatment-responsive and 5 treatment-resistant participants were incorrectly classified (sensitivity = 67%, specificity = 94%). (**C**) shows the regression curve for the two independent variables that added significantly to the model; right amygdala and left pallidum activity. Points on the curve show the predicted probability of treatment resistance based on the parameter estimates from visit A and the points at y = 0 (treatment responsive) and y = 1 (treatment-resistant) show the actual classifications of participants.

The logistic regression model which included beta scores from right amygdala, left pallidum and mPFC (all from emotional condition), plus ACC glutamate levels and behaviour (ideal choices) measured from visit A described 61% of the variation in treatment resistant status (Nagelkerke R^2^ = 0.61) (Fig. 4A) and correctly identified 86% of cases. The model showed a sensitivity of 67% (10/15 treatment-resistant correctly classified) and a specificity of 94% (33/35 treatment-responsive correctly classified) (Fig. 4B). Right amygdala (β = − 0.67, *p* < 0.01) and left pallidum (β = − 1.51, *p* < 0.01) activity at visit A both added significantly to the model where lower activity in these regions was associated with an increased likelihood of being treatment-resistant (Fig. 4C). Activity in mPFC, behavioural performance (overall proportion of ideal choices) and glutamate levels at visit A were not significant predictors.

## Discussion

To our knowledge, this study is the first to examine the longitudinal course of cognitive control related functional deficits during an emotion-yoked reward learning task in relation to treatment response in individuals with early psychosis. We provide evidence that deterioration in mPFC activity related to cognitive control function is a factor underlying the development of treatment-resistant psychosis. Compared to treatment-responsive participants, treatment-resistant participants showed altered activity in right amygdala (decision phase) and left pallidum (feedback phase) at baseline (1–2 years illness duration) which increased over time (one year) and was accompanied by a decrease in mPFC activity (feedback phase) over time. These findings were evident during the emotional condition (high cognitive demand) and reward learning behaviour in the emotional condition was also impaired in the resistant participants. Only amygdala and pallidum activity at baseline was predictive of treatment resistant status at one-year follow-up, suggesting declining cognitive control (mPFC) function over an initial dysfunction within a social-emotional network were contributors of treatment resistance. In contrast, recruitment of mPFC and a decline in pallidum activity was evident in treatment responsive participants, suggesting effective mPFC related cognitive control function was important for control of symptoms. Finally, there was no evidence for a difference in glutamate levels between groups or over time, however, cognitive control activity in mPFC was uncoupled to ACC glutamate levels in resistant participants at follow-up. This could suggest that cognitive control function is unsupported by glutamate signaling in treatment resistance and warrants further investigation.

Previous studies have emphasized the role of poor cognitive control and prefrontal cortex dysfunction or ‘hypofrontality’ in schizophrenia. The literature is unclear as to whether these deficits relate to treatment resistance and if they are neurodevelopmental (occur before psychosis onset) or neurodegenerative (deteriorate over time). In early psychosis, one study with a similar longitudinal design showed reduced cognitive control activity in dorsolateral prefrontal cortex (DLPFC) at baseline using a typical cognitive control task (AX-Continuous Performance Task). However the authors reported that DLPFC activity followed a similar developmental trajectory after 1–2 years compared to healthy controls—supportive of a neurodevelopmental abnormality^23^. Interestingly, our previous study in the same current sample showed that treatment-resistant participants had altered cognitive control behaviour (as measured by a slower Stroop effect reaction time) compared to treatment-responsive participants that was present at baseline and did not change over time^12^. Moreover, participants with greater severity of positive symptoms at baseline showed altered activity in a subcortical network (left amygdala, left pallidum, left thalamus) and reduced activity in a different cognitive control region (Supplementary Motor Area) compared to participants with lower severity of positive symptoms^10^. This suggests some cognitive control dysfunction is observed at baseline but contrary to previous findings, baseline mPFC cognitive control function was not predictive of treatment resistance but deteriorated over time (although we did not have longitudinal data from healthy controls as a comparison). Most other studies of treatment response to date have studied neural responses to acute administration of antipsychotic medication (e.g., after 3–12 weeks)^24^. Cadena et al.^8^ showed that baseline ACC activity was not significantly different between people with psychosis and controls but an increase in ACC activity over a 6-week course of risperidone was correlated with an improvement in psychotic symptoms. Similar to the current findings, this suggests improvement in prefrontal cortex function is important for treatment response. Interestingly, the same authors reported that greater functional connectivity between ACC and putamen at baseline predicted subsequent better treatment response^9^. We also reported enhanced top-down connectivity by the ACC in treatment-responsive people with chronic schizophrenia^7^. Prefrontal cortex connectivity at baseline may therefore be important for subsequent cognitive control function and treatment response and this warrants further investigation.

Deterioration in mPFC function was preceded by dysfunction within right amygdala and left pallidum at baseline—components of a social-emotional network. Activity within these two regions was also different between early psychosis participants with high and low positive symptoms in our earlier study^10^. Poor emotion and salience processing of facial expressions has been widely reported in psychosis^25–27^ with particular abnormalities reported in amygdala^27^, as well as pallidum^28^. Poor cognitive control over emotion has also been identified as a potential risk factor for psychosis^29^ suggesting affective deficits may be present before psychosis onset. Here, altered activity in amygdala was present in both emotional and neutral conditions and therefore may represent a general deficit in emotion processing or allocation of salience to facial expressions when decision-making. Blunted activity in both right amygdala and left pallidum at baseline was predictive of treatment resistance (with reasonable sensitivity and high specificity) where resistant participants showed ineffective modulation of this response over time (increase in activity). Similar to a previous study^8^, this suggests baseline functioning of subcortical regions is important for treatment response and may even be a ‘primer’ for effective cognitive control.

Glutamate levels in ACC were not significantly increased in treatment-resistant participants compared to treatment-responsive, contrary to some studies^4,6,30,31^ but similar to others^32,33^. Instead, better cognitive control function (higher mPFC activity) was related to lower glutamate levels at follow-up in treatment-responsive participants, but no such relationship existed in treatment-resistant participants. This finding is similar to our previous study in people with chronic schizophrenia where ACC connectivity was uncoupled (unrelated) to ACC glutamate levels in the treatment-resistant group only^7^. But another study reported lower ACC glutamate predicted poorer cognitive control abilities in schizophrenia, showing an opposite relationship to ours^34^. In a recent mega-analysis of MR spectroscopy studies in schizophrenia, Merritt et al.^35^ reported that higher glutamate levels in medial frontal cortex were associated with greater severity of positive symptoms and lower medication dose suggesting effective treatment reduces glutamate levels (considered to be neurotoxic in excess). Here, we showed that treatment response may not be directly related to static glutamate levels but could be related to effective glutamatergic signaling supporting cognitive control function, although the causal nature of this relationship could not be determined in this study. Additionally, there was larger variability in mPFC activity at visit C in the treatment-resistant group. It is unknown why this was but could be due to other factors associated with treatment resistance e.g., genetic variability^36^, a longer duration of untreated psychosis or greater severity of both positive and negative symptoms^37^, or a smaller sample size. Together these findings could point towards a cognitive control dysfunction with a potential non-dopaminergic factor important for understanding treatment resistance in early psychosis which should be investigated further. Indeed, other studies have suggested that glutamate could be a candidate target for novel antipsychotic medications. For example, early data suggest clinical efficacy of novel glutamatergic agents^38^.

### Limitations

First, whilst our study followed on from an examination of early psychosis participants versus healthy controls^10^, follow-up data was not collected from healthy controls. Therefore, the longitudinal changes reported between patient groups cannot be directly compared to ‘normal’ changes in cognitive control function over time. Second, patient medication was not controlled, and participants were taking different types and combinations of antipsychotics. The aim of the study was to follow participants naturally over a period of one year to understand brain function in participants that were categorized into a treatment-resistant ‘phenotype’, regardless of medication course. However, the effects of different medications on the current findings cannot be disentangled. For example, medications with low dopaminergic receptor affinity and high serotonergic receptor affinity are associated with higher prefrontal cortex activation^39^ and so the effects of medications should be taken into consideration in larger future studies. Third, our analyses were restricted to the participants that were followed up at visit C. Although the percentage drop out from visits A to C was greater in participants with greater severity of positive symptoms (18/67, 27%) than lower severity of positive symptoms (4/30, 13%), the reasons for the drop-out are unclear and may reflect worsening symptoms or other health issues that introduces (unavoidable) bias to the sample. Moreover the treatment-resistant sample size was modest—reflecting the difficulty in recruiting, retaining and classifying treatment-resistant psychosis participants. Replication studies are therefore needed in future. Fourth, whilst we used an emotion-yoked reward learning task that required cognitive control to study cognitive control function here, as in our other studies, this was not a ‘typical’ cognitive control task and other processes (e.g., emotion processing, reward processing) may have confounded the results. Future longitudinal studies using e.g., Stroop tasks would be useful to aid interpretation. Finally, whilst the current study aimed to investigate a potential non-dopaminergic (glutamatergic) mechanism associated with cognitive control dysfunction in treatment resistant psychosis, we did not directly or indirectly measure dopaminergic function. Our previous study using the same emotion-yoked reward learning task showed that treatment-resistant participants had intact RPE-related activity (an indirect measure of dopaminergic function). However, future studies measuring dopaminergic and glutamatergic function, alongside cognitive control function, are needed. Similarly, glutamate measured via MRS spectroscopy represents an indirect measure of static concentrations of the metabolite in a relatively large area of the brain (ACC). Future studies measuring glutamate directly or dynamically during a cognitive control task would be useful for elucidating the effect further.

## Conclusion

Results suggest that baseline alterations in a social-emotional network followed by a deterioration in cognitive control related mPFC function over time may represent a factor underlying the development of treatment resistance in early psychosis. An uncoupling between cognitive control related mPFC function and glutamate levels was also observed. The findings could help to explain why currently available first-line antipsychotics (that primarily target dopamine receptors in the brain and have little impact on cognitive symptoms) are ineffective in treating symptoms in one third of people with schizophrenia. The findings suggest a potential avenue for new medications and alternative treatment strategies e.g., giving clozapine earlier^2^ or stimulating cognition using transcranial direct current stimulation (TDCS)^40^. Future studies that investigate the utility of modulating cognitive control mechanisms or glutamate function in schizophrenia are needed.

### Supplementary Information

Supplementary Information.

## Data Availability

The data supporting this article is not openly available due to ethical and regulatory restrictions but may be available from the corresponding author on reasonable request.
