# Supplementary material for: Deterioration in cognitive control related mPFC function underlying development of treatment resistance in early psychosis
Source: Sci Rep. 2024 Jun 6;14:12985. doi: 10.1038/s41598-024-63474-1 (PMC11153613; doi:10.1038/s41598-024-63474-1)
Supplement: Supplementary file 1 — Supplementary Information. [file 41598_2024_63474_MOESM1_ESM.docx]

**Supplementary materials and methods**

***Participants***

PANSS and SOFA were assessed by a trained professional and medication trials and adherence was assessed via clinical reports and the patient’s clinical care team. Participants were excluded if they reported a history of neurological illness, current major physical illness, diagnosed drug dependency over the past six months or a contraindication for MRI. Most participants (47/50) were medicated at visit A except 3 treatment-responsive participants and this reduced to 31/50 participants at visit C where 14 treatment-responsive participants were discharged/not on medication and 5 participants (3 treatment-responsive, 2 treatment-resistant) had an unknown medication status. Considering this, the groups did not significantly differ in medication dose (chlorpromazine equivalent dosage) or medication adherence.

***Reward learning task***

A fixation cross was presented (1000 ms) followed by two faces (side-by-side) where participants selected one of the faces using a button box and right index finger. Participants then received feedback (either ‘You win 10p!’ or ‘You lose’) on the screen for 1500 ms. Participants were asked to choose between two faces (side-by-side) on each trial and were required to learn - over a series of 30 iterative trials (per block) - which of the two faces was associated with a higher probability of reward (reward contingencies were 60%/40%). Participants received additional payment based on their performance. Combinations of identities and reward contingencies were counterbalanced across blocks and participants^2^. The task took approximately 15 minutes to complete.

***Imaging data analysis***

First, the T1 structural and functional images were skull-stripped and the origin of the images was manually reset over the anterior commissure. The functional images were realigned to correct for the effects of head motion, co-registered to the structural images and normalised to Montreal Neurological Institute (MNI) space. The functional images were then filtered using a temporal high pass filter of 100 s and spatially smoothed using a 6mm FWHM Gaussian kernel.

A general linear model was used in SPM to analyse the data. Similar to our previous paper (Horne et al 2021b), the first-level analysis included 6 regressors that modelled the three phases of the task (face presentation, decision (button press) and feedback) separately for the two conditions; emotional and neutral. Each regressor was modelled as an event with a delta function (duration = 0) and was convolved with a canonical haemodynamic response function (hrf) and its temporal derivative. Six standard subject-specific motion parameters were estimated and added as regressors of no interest to the model to control for head motion.

For each participant, first-level analyses were constructed for each visit (A, C) and each condition (emotional, neutral) where the decision and feedback phases were examined. Since we were primarily interested in the group x visit interaction, 4 contrasts of interest were constructed: 1) Emotional decisions (visit A – visit C), 2) Emotional feedback (visit A – C), 3) Neutral decisions (visit A – visit C), and 4) Neutral feedback (visit A – visit C).

The 4 contrasts of interest were then submitted to separate mixed-effects analyses at the group-level. Two independent sample t-tests were used to compare activity between treatment-resistant and treatment-responsive participants (giving the group x visit interaction). As the groups did not significantly differ in age, sex, illness duration or medication dose, these were not added as covariates to maintain statistical power.

***Regions of interest***

All ROIs were binary masks that were anatomically defined using the probabilistic Harvard Oxford Subcortical Structural atlas or cortical atlas (for ACC) thresholded at 30%. For the decision phase, bilateral amygdala was chosen as a key emotional processing region along with the ACC. The ACC was also used as an ROI during the decision and feedback phases as it is involved in both reward prediction and cognitive control ^3,4^. The bilateral striatum plus pallidum and thalamus were used as an ROI for the feedback phase because of the key role these structures play in reward learning^5^.

A cluster defining threshold of p < 0.001 uncorrected was used to perform separate ROI analyses and significant effects are reported if they survive small volume correction (SVC) with a peak level threshold of p < 0.05 FWE-corrected. Complementary whole-brain analyses were also conducted using a cluster-level FWE-corrected threshold of p < 0.05 (cluster defining threshold of p < 0.001). All significant effects are reported from the coordinates of the peak activation in MNI space (XYZ).

***Glutamate***

First, a mixed-model ANOVA was conducted to test for a significant group x visit interaction on glutamate levels since there is some evidence that glutamate may be elevated in ACC in treatment-resistant participants ^7,8^. Then, we tested whether glutamate was related to differences in mPFC functional activity identified between treatment-responsive and resistant participants by 1) adding glutamate (from visit A) as a parametric covariate to the GLM to test for a relationship (whole-brain analysis) between initial glutamate levels and change in PFC activity over time during emotional feedback (one-sample t-test), and 2) extracting the parameter estimates at visit C from the peak voxel of PFC activity that showed a significant group x visit interaction and correlating these with glutamate levels at visit C. Parameter estimates were extracted using the marsbar toolbox in SPM and Spearman’s correlations (as glutamate levels were not normally distributed) were conducted across all participants and within groups separately. Analyses were restricted to the ACC and mPFC to ensure anatomical overlap.

**Table S1**

1H-MRS metabolite concentrations in the Anterior Cingulate Cortex in Treatment-Responsive and Treatment-Resistant participants.


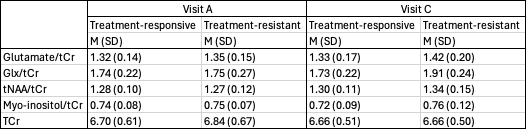


Note: Means (M) and Standard Deviations (SD) for metabolite concentrations. tCr, total Creatine (Creatine + Phosphocreatine); Glx, Glutamate + Glutamine; total NAA (N-acetyl aspartate + N-acetylaspartylglutamate).

**Table S2**

1H-MRS Spectra Quality Measures (mean (SD)) in the Anterior Cingulate Cortex in Treatment-Responsive and Treatment-Resistant participants.


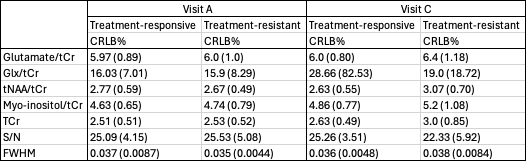


**Note:** Means (M) and Standard Deviations (SD) for Cramer-Rao Lower Bounds (CRLB%). tCr, total Creatine (Creatine + Phosphocreatine); Glx, Glutamate + Glutamine; tNAA, total NAA (N-acetyl aspartate + N-acetylaspartylglutamate). S/N, signal-to-noise ratio; FWHM, full-width-half-maximum (ppm). Sequence is not optimized for Glx.

***Prediction***

A logistic regression was performed to test whether functional activity at visit A predicted treatment response status at visit C. Parameter estimates from three clusters of activation showing a significant group x visit interaction (right amygdala, left pallidum, mPFC) were extracted at visit A using marsbar. Analyses were performed in SPSS where parameter estimates from the three regions plus glutamate levels and behavioural performance (overall proportion of ideal choices) at visit A were entered as predictor variables and treatment resistance status as the output variable.

**Additional figures**


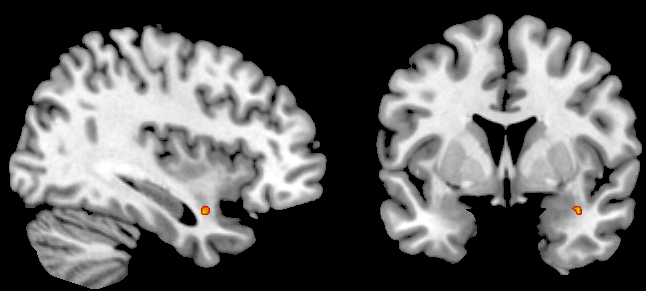


Figure S1. Significant group x visit interaction in Right Amygdala during neutral decisions.

**
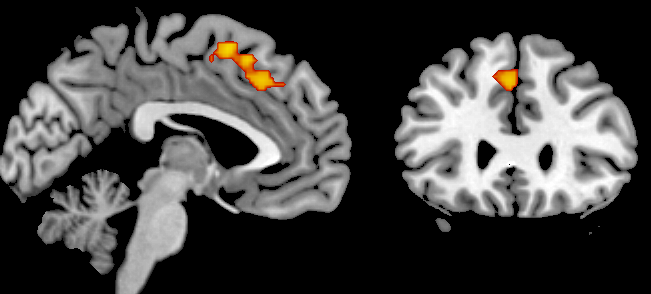
**

Figure S2. Significant group x visit interaction in mPFC/SMA during the emotional vs. neutral feedback.

**Additional references**

1. Rybarczyk B. Social and Occupational Functioning Assessment Scale (SOFAS). In: Kreutzer JS, DeLuca J, Caplan B, eds. *Encyclopedia of Clinical Neuropsychology*. Springer New York; 2011:2313-2313.

2. Evans S, Shergill SS, Chouhan V, Bristow E, Collier T, Averbeck BB. Patients with schizophrenia show increased aversion to angry faces in an associative learning task. *Psychol Med*. Jul 2011;41(7):1471-9. doi:10.1017/s0033291710001960

3. Kerns JG, Cohen JD, MacDonald AW, 3rd, Cho RY, Stenger VA, Carter CS. Anterior cingulate conflict monitoring and adjustments in control. *Science*. Feb 13 2004;303(5660):1023-6. doi:10.1126/science.1089910

4. Brown JW, Braver TS. Learned predictions of error likelihood in the anterior cingulate cortex. *Science*. Feb 18 2005;307(5712):1118-21. doi:10.1126/science.1105783

5. Schultz W. Reward functions of the basal ganglia. *J Neural Transm (Vienna)*. 2016;123(7):679-693. doi:10.1007/s00702-016-1510-0

6. Provencher SW. Automatic quantitation of localized in vivo 1H spectra with LCModel. *NMR Biomed*. Jun 2001;14(4):260-4.

7. Demjaha A, Egerton A, Murray RM, et al. Antipsychotic treatment resistance in schizophrenia associated with elevated glutamate levels but normal dopamine function. *Biol Psychiatry*. Mar 1 2014;75(5):e11-3. doi:10.1016/j.biopsych.2013.06.011

8. Mouchlianitis E, Bloomfield MA, Law V, et al. Treatment-Resistant Schizophrenia Patients Show Elevated Anterior Cingulate Cortex Glutamate Compared to Treatment-Responsive. *Schizophrenia bulletin*. May 2016;42(3):744-52. doi:10.1093/schbul/sbv151
